# The Effects of Infant Massage Therapy on Preterm Neonatal Outcomes: A Clinical Trial

**DOI:** 10.1155/ijpe/2451284

**Published:** 2025-02-27

**Authors:** Jiranun Weerakul, Yasinee Apiraknapanon, Mathayan Sanjaiban, Suneera Intasen, Supattra Tipsuwan, Mattana Bhumipraphat

**Affiliations:** ^1^Pediatrics Department, Faculty of Medicine, Naresuan University, Phitsanulok, Thailand; ^2^Nursing Department, Naresuan University Hospital, Phitsanulok, Thailand; ^3^Department of Physical Therapy, Faculty of Allied Health Sciences, Naresuan University, Phitsanulok, Thailand

**Keywords:** baby massage, outcomes, preterm

## Abstract

**Background:** Preterm infants are at risk of impaired growth and prolonged hospitalization due to their immature organ systems and treatment complications. Infant massage therapy, as a nonpharmacological intervention, has been recognized as a potential strategy to mitigate these challenges. This study is aimed at evaluating the efficacy of infant massage therapy in comparison to standard care.

**Method:** Participants were allocated into two groups: the intervention group (infants who received massage therapy) and the control group (infants who received standard care). Inclusion criteria were newborn infants with a postmenstrual age (PMA) of 30–36 + 6 weeks and a body weight of ≥ 1500 g. Infants with congenital anomalies, such as heart disease, congestive heart failure, lung and airway anomalies, other congenital anomalies, endotracheal intubation, and unstable vital signs, were excluded. The intervention group received massage therapy for 14 consecutive days, with sessions lasting 15 min each. Metrics for growth and length of hospital stay were collected.

**Results**: Infants born with very low birth weight (VLBW) who received massage therapy exhibited significant weight gain, with an average increase of 63.04 g per day (95% confidence interval (CI): 11.72–114.35), significantly higher than the control group (*p* = 0.02). Additionally, the duration from birth to achieving full feeding was significantly reduced in the intervention group compared to the control group, with durations of 18.75 (± 10.86) and 41.88 (± 23.09) days, respectively (*p* = 0.01).

**Conclusion**: Infant massage therapy significantly enhances weight gain and reduces the time to achieve full feeding in VLBW preterm infants. Moderate-pressure massage is recommended for stable VLBW infants in neonatal intensive care units (NICUs).

## 1. Introduction

The survival rates of premature infants have significantly improved with the introduction of surfactant therapy as the standard treatment for respiratory distress syndrome. The primary goal of quality care for preterm neonates extends beyond ensuring survival; it encompasses fostering long-term growth, achieving age-appropriate developmental milestones, optimizing neurological function, and minimizing potential sequelae associated with prematurity. Sensory deprivation in neonates, characterized by the absence of skin-to-skin contact and interoceptive cues during incubator care in the neonatal intensive care unit (NICU), can trigger physiological stress responses. Studies have demonstrated that tactile and kinesthetic stimulation is associated with improved weight gain, enhanced neurodevelopmental outcomes, and reduced stress responses in preterm neonates [1–3].

Infant massage, a tradition in many cultures that involves structured skin-to-skin touch, offers several benefits for newborns, although the duration, intensity, extent, use of oil, and parental involvement vary across cultures [3]. Practiced in approximately 38% of US NICUs, research suggests that infant massage can be particularly beneficial for babies in NICUs, with premature infants showing significant physical and neurological improvements, weight gain, shorter hospital stays, and fewer postnatal complications [1, 4–6].

Premature infants receiving massage therapy show remarkable physical and neurological progress, enhanced growth, and development [4]. Moderate-pressure massage, in particular, has been found to offer superior advantages over light-pressure massage. Infants subjected to moderate-pressure massage exhibit improved growth and development, as well as decreased heart rate when stressed [7–9].

Moderate-pressure massage is characterized as a technique that applies firmer pressure than light touch but is less intense than deep-tissue massage. This technique targets the deeper layers of muscle and fascia, providing several distinct benefits. Specifically, moderate-pressure massage enhances relaxation and stress reduction in preterm infants by stimulating pressure receptors, thereby increasing vagal activity, and facilitating a shift towards the parasympathetic nervous system. This promotes relaxation and stress alleviation, in addition to triggering the release of endorphins, natural painkillers that mitigate pain and discomfort [8].

This study is aimed at comparing the growth parameters and lengths of hospital stay between preterm infants receiving moderate-pressure massage therapy and those receiving standard treatment.

## 2. Material and Methods

This prospective, randomized controlled trial involved preterm infants in the NICU and was conducted between February 2021 and May 2023. Inclusion criteria included newborn infants with a postmenstrual age (PMA) of 30–36 + 6 weeks and a body weight of ≥ 1500 g. Infants with birth defects such as congenital heart disease, congestive heart failure, congenital lung and airway anomalies, other congenital anomalies, those requiring endotracheal intubation, and those with unstable vital signs were excluded from this study.

After explaining the study's objectives and obtaining informed consent from the parents, neonates were randomly allocated into two groups using a block randomization method: The intervention group, which consisted of neonates receiving massage therapy, and the control group, which comprised of infants receiving standard care. The intervention group underwent massage therapy for 14 consecutive days, receiving one 15-min session daily administered by a NICU nurse. Conversely, the control group received standard care without the inclusion of massage therapy. Data collection was divided into two parts: The first consisted of collecting demographic data about mothers and infants, while the second part involved measurements of weight, length, head circumference, and length of stay in both the NICU and the hospital.

### 2.1. Intervention

Massage therapy was initiated at a corrected gestational age of over 30 weeks and continued for 14 consecutive days. Each session involved 15 min of moderate-pressure tactile stimulation, during which the infant was placed in a supine and prone position. Moderate-pressure strokes were applied using the ventral and dorsal parts of the fingers across the infant's head, neck, shoulders, buttocks, and limbs. Throughout the therapy, close observation for signs of distress (e.g., crying, irritability, and cyanosis) was conducted.

Body parameters, including body weight, length, and head circumference, were assessed for both groups at baseline (Day 0) and daily across the 14 days. In cases where a participant was discharged before completing the 14-day regimen, their data were collected for intention-to-treat analysis.

The study was approved by the Institutional Review Board of Naresuan University (IRB No. P3-0002/2564).

### 2.2. Statistical Analysis

The study sample size was calculated based on Massaro et al. [1]. The average daily weight gain over the study period was 28.9 ± 1 g for the control group and 30 ± 1.2 g for the massage with exercise (M/KS) groups. This study determined the sample size using an alpha of 0.05 and a beta of 0.20, calculated for two separate tests. Accounting for a dropout rate of 20%, the required sample size was established at 20 participants per group, based on an initial calculation for 16 individuals per group.

Descriptive statistics for continuous data was employed to assess the dataset's distribution. Data following a normal distribution were summarized using the mean ± standard deviation. In instances where data deviated from normality, findings were presented using the median and interquartile range (IQR). Additionally, data on group variables were presented using frequency and percentage distributions.

The selection of statistical tests for comparing differences between the two groups was contingent upon the distribution of the continuous data. For data adhering to a normal distribution, the independent *t*-test was utilized. The chi-square test, alongside a mixed-effects model for linear regression, was applied to evaluate differences in group variables and to analyze body weight at birth in infants weighing < 1500 g. All analyses were conducted with a set statistical significance level of 0.05.

## 3. Results

**Figure 1:**
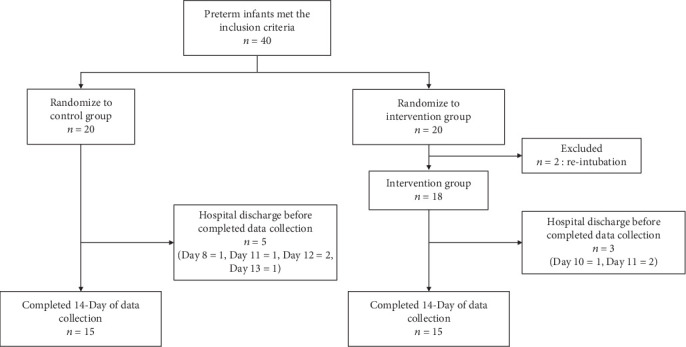
Summary of preterm neonates in the study.

Among the eligible 38 preterm infants, 20 were randomly assigned to the control group (infants who received standard care) and 18 to the intervention group (infants who received massage therapy) (Figure 1). Maternal demographic data revealed no statistically significant differences between the two groups. The massage intervention group exhibited fewer days of intubation and parenteral nutrition than the control group, a statistically significant difference (Table 1).

Feeding outcomes, such as age at full feeding (via OG tube), the initiation age for oral feeding, and the age at full oral feeding, did not significantly differ between the two groups. However, a subgroup analysis indicated that very low birth weight (VLBW) infants, with birth body weight ≤ 1500 g, in the intervention group achieved full feeding on average 23 days earlier than those in the control group, with a statistically significant difference (*p* = 0.01). The age at full oral feeding for VLBW infants in the intervention group was approximately 15 days earlier than in the control group, though this difference was not statistically significant (*p* = 0.31) (Table 2).

VLBW infants in the intervention group had a shorter length of NICU stay by an average of 28.10 days, a statistically significant difference (*p* = 0.01). Similarly, low birth weight infants in the intervention group were discharged from the hospital 29.98 days earlier than those in the control group (*p* = 0.12) (Table 2).

Among all participants, there was no significant difference in body weight, length, and head circumference between the two groups before intervention.

**Figure 2:**
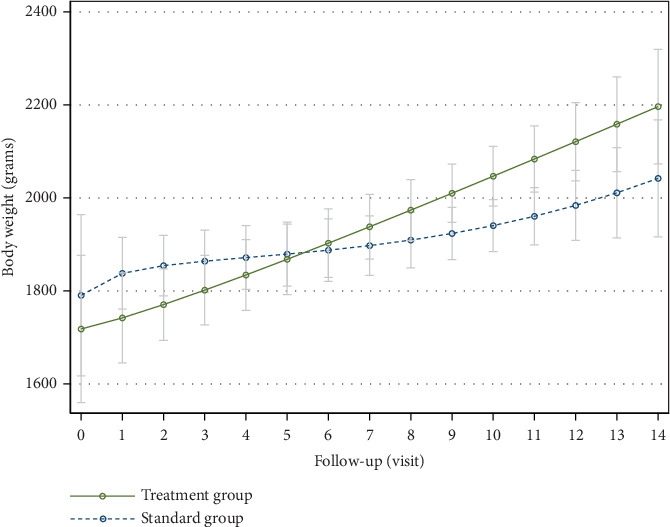
Mixed-effects model for linear regression of body weight in infants with birth weight < 1500 g.

The analysis of weight gain over the 14 days, using a mixed-effects model for linear regression, revealed that infants born with VLBW in the intervention group experienced significant weight gain. Each day of baby massage was associated with an increase in body weight of 63.04 g (95% confidence interval (CI): 11.72–114.35), remarkably higher than that observed in the control group, with statistical significance (*p* = 0.02) (Figure 2).

## 4. Discussion

This study enrolled 38 preterm infants. Maternal demographic data and infant baseline characteristics showed no significant differences between the intervention group and the control group, except for the duration of intubation and parenteral nutrition, which was significantly shorter in the intervention group.

In the subgroup of VLBW infants, the age at full feeding was significantly reduced in the intervention group compared to the control group (*p* = 0.01). This observation supports the hypothesis that moderate-pressure massage might mitigate stress and dampen sympathetic nervous system activity, thereby favorably impacting gastrointestinal muscle and mucosal secretion [8, 10]. Such mechanisms could facilitate improved feeding in VLBW infants, who are subject to greater physical stress than infants with low birth weights.

The initiation of oral feeding and the age at achieving full oral feeding did not differ between the two groups. This consistency can be attributed to the implementation of a nonnutritive suckling program at the PMA of 34 weeks, which forms part of the standard care protocol in our hospital for both groups.

The primary outcomes of this study were growth parameters and the length of hospital stay. Our findings indicated no significant difference in weight gain between the intervention group and the control group, which is consistent with the results of Mollà-Casanova, et al. who found that massage therapy alone did not impact weight gain in their study population [11]. However, prior research has suggested a potentially beneficial effect of massage therapy on weight gain in premature infants [5, 12–14]. Infants with VLBW who received moderate-pressure massage exhibited a significant increase in weight gain compared to those who received standard care. A mixed-effects model for linear regression on infants with birth body weight <1500 g revealed that daily moderate-pressure massage resulted in an average weight gain of 63.04 g (95% CI: 11.72–114.35), significantly surpassing the control group (*p* = 0.016). This finding contradicts the observations that low birth weight infants receiving moderate-pressure massage showed a positive impact on weight gain, though it was not statistically significant in the VLBW subgroup [9]. Our results corroborate those of Rad et al., who reported a notable increase in the average weight of VLBW infants in the baby massage group from the fifth day of a thrice-daily intervention for a week [15]. Implementing baby massage once daily for 14 days in our study yielded similar outcomes to those of Rad et al. [15]. Further evidence supports the positive effect of tactile and kinesthetic stimulation on weight gain in preterm infants [16], highlighting the advantage of baby massage in promoting weight gain in VLBW infants over standard care.

NICU stays for VLBW infants were significantly shorter in the intervention group, supporting studies by Field and Hernandez-Reif and Rad et al. [5, 15]. Although no significant differences were found in the overall length of hospital stays between the groups, the observed reduction of 21.55 days for the intervention group is clinically significant, indicating potential for quicker recovery and reduced hospital costs [13, 16–18].

This study is subject to several limitations. Firstly, it did not account for various factors that could influence infant weight gain, such as total fluid volume and caloric intake. Generally, preterm care aims to maximize feeding volume to 160 mL/kg/day with a standard caloric density of 24 kcal/oz in fortified breast milk or premature formula for all preterm infants. High caloric density is reserved for specific conditions like volume-sensitive bronchopulmonary dysplasia, with a general target for total caloric intake being 120–130 kcal/kg/day for every preterm patient. Future research should explore these areas more thoroughly, enhancing data collection and implementing standard feeding protocols.

Lastly, the benefits of baby massage, such as improvements in oxygen saturation and mother–infant attachment, have been highlighted in systematic reviews and meta-analyses of randomized controlled trials [19, 20]. Due to restrictions during the COVID-19 pandemic, our study was unable to allow mothers to participate in massage therapy with their children, and thus, data on maternal relationships and satisfaction with massage therapy were not collected. Despite this, no adverse events related to desaturation were observed during massage sessions. Future research should include maternal participation in the massage process to gather more comprehensive data. It may also benefit from focusing on targeted populations, such as infants with VLBW, employing larger sample sizes, and investigating additional potential advantages.

## 5. Conclusion

Moderate-pressure massage is a cost-effective, noninvasive intervention free from adverse health effects. Also, moderate-pressure massages have shown positive effects on weight gain and reduced lengths of hospital stay in infants with VLBW. Therefore, incorporating moderate-pressure massage into the standard care of preterm infants, who continue to represent a significant national health challenge, is recommended, particularly for those with a body weight of ≤ 1500 g.

## Figures and Tables

**Table 1:** Demographic data.

	**Intervention group (** **n** = 18**, mean ± SD)**	**Control group (** **n** = 20**, mean ± SD)**	**p** ** value**
Maternal age (years)	26 ± 7.43	27.75 ± 6.57	0.77
Presence of maternal underlying disease	5 (27.78%)	4 (20%)	0.71
Gestational age at birth (weeks)	30.89 ± 2.30	30.65 ± 3.60	0.81
Infant male sex	11 (61.11%)	13 (65%)	0.54
Birth body weight (g)	1463.90 ± 366.41	1533.50 ± 592.83	0.43
Birth body weight (g)			
Below 1000	2 (11.11%)	6 (30%)	0.04∗
1001–1500	8 (44.44%)	2 (10%)	
1501–2500	8 (44.44%)	12 (60%)	
Apgar score at 1 min	6.8 ± 1.9	6.2 ± 2.5	0.39
Apgar score at 5 min	8.72 ± 1.07	8.15 ± 2.11	0.31
Vaginal delivery	6 (33.33%)	5 (25%)	0.72
Respiratory disease			0.32
RDS	7 (38.89%)	12 (60%)	
TTNB	8 (44.44%)	6 (30%)	
Congenital pneumonia	3 (16.67%)	1 (5%)	
Intubation (days)	2.5 (1.5, 9.5) *n* = 8	28.5 (6, 45) *n* = 8	0.01∗
Present history of necrotizing enterocolitis	3 (16.67%)	7 (35%)	0.28
Duration of NPO before initiating first feeding (days)	3.88 ± 6.23 *n* = 17	3.9 ± 6.69 *n* = 20	0.1
Total TPN duration (days)	9.56 ± 7.39 *n* = 16	24.06 ± 19.56 *n* = 17	0.01∗

∗*p* ≤ 0.05.

**Table 2:** Outcomes of infant feeding and length of hospital stay.

	**Intervention group (mean ± SD)**	**Control group (mean ± SD)**	**p** ** value**
Age at full feeding (days)	13.59 ± 8.73 *n* = 17	24.4 ± 21.83 *n* = 20	0.06
Birth body weight ≤ 1500 g	17.20 ± 9.67 *n* = 10	41.88 (± 23.09) *n* = 8	0.01∗
Birth body weight > 1500 g	8.43 ± 3.21) *n* = 7	12.75 ± 10.65 *n* = 12	0.32
Age at initiation of oral feeding (days)	21.94 ± 16.23 *n* = 18	24.3 ± 25.87 *n* = 20	0.74
Age at full oral feeding (days)	40.28 ± 26.68 *n* = 18	36.05 ± 36.97 *n* = 19^†^	0.69
Birth body weight ≤ 1500 g	57.2 ± 23.82 *n* = 10	72.43 ± 36.09 *n* = 7	0.31
Birth body weight > 1500 g	19.13 ± 8.85 *n* = 8	14.83 ± 13.88 *n* = 12	0.45
Weight change from 0 to 14 days protocol (g)	421.33 (115.97) *n* = 15	403 (137.67) *n* = 15	0.70
Length change from 0-14 days protocol (cm)	2.63 ± 1.52 *n* = 15	2.73 ± 1.56 *n* = 15	0.86
Head circumference change from 0 to 14 days protocol (cm)	1.83 ± 0.62 *n* = 15	1.67 ± 0.77 *n* = 15	0.52
NICU length of stay (days)	30.39 ± 16.72 *n* = 18	35.35 ± 33.80 n =20	0.58
Birth body weight ≤ 1500 g	40.4 ± 13.39 *n* = 10	68.5 ± 28.65 *n* = 8	0.01∗
Birth body weight > 1500 g	17.88 ± 11.23 *n* = 8	13.25 ± 10.91 *n* = 12	0.37
Length of hospital stay (days)	53.83 ± 30.10 *n* = 18	56.9 ± 51.33 *n* = 20	0.83
Birth body weight ≤ 1500 g	73.90 ± 24.51 *n* = 10	103.88 ± 51.01 *n* = 8	0.12
Birth body weight > 1500 g	28.75 ± 11.57 *n* = 8	25.58 ± 14.83 *n* = 12	0.62

^†^One participant has been referred out before full oral feeding.

∗*p* ≤ 0.05.

## Data Availability

The data that support the findings of this study are available on request from the corresponding author. The data are not publicly available due to privacy or ethical restrictions.
